# The Role of Genetic Testing in Patients with Heritable Thoracic Aortic Diseases

**DOI:** 10.3390/diagnostics13040772

**Published:** 2023-02-17

**Authors:** Emanuele Monda, Michele Lioncino, Federica Verrillo, Marta Rubino, Martina Caiazza, Alfredo Mauriello, Natale Guarnaccia, Adelaide Fusco, Annapaola Cirillo, Simona Covino, Ippolita Altobelli, Gaetano Diana, Giuseppe Palmiero, Francesca Dongiglio, Francesco Natale, Arturo Cesaro, Eduardo Bossone, Maria Giovanna Russo, Paolo Calabrò, Giuseppe Limongelli

**Affiliations:** 1 Inherited and Rare Cardiovascular Diseases, Department of Translational Medical Sciences, University of Campania “Luigi Vanvitelli”, Monaldi Hospital, Via L. Bianchi, 80131 Naples, Italy; 2 Department of Public Health, University of Naples “Federico II”, Via L. Pansini, 80131 Naples, Italy; 3 Institute of Cardiovascular Sciences, University College of London and St. Bartholomew’s Hospital, London WC1E 6DD, UK

**Keywords:** aortic disease, Marfan syndrome, genetics, prognosis

## Abstract

Heritable thoracic aortic disease (HTAD) is a term used to define a large group of disorders characterized by the occurrence of aortic events, mainly represented by aneurysm or dissection. These events generally involve the ascending aorta, although the involvement of other districts of the aorta or peripheral vessels may occur. HTAD can be classified as non-syndromic if the disorder is limited to the aorta, and syndromic when associated with extra-aortic features. About 20–25% of patients with non-syndromic HTAD exhibit a family history of aortic disease. Thus, a careful clinical evaluation of the proband and the first-degree family members is required to differentiate familial and sporadic cases. Genetic testing is essential since it allows confirmation of the etiological diagnosis of HTAD (particularly in patients with a significant family history) and may guide family screening. In addition, genetic diagnosis significantly impacts patients’ management since the different conditions significantly differ with respect to natural history and treatment strategies. The prognosis in all HTADs is determined by the progressive dilation of the aorta, potentially leading to acute aortic events, such as dissection or rupture. Moreover, the prognosis varies according to the underlying genetic mutations. This review aims to describe the clinical characteristics and natural history of the most common HTADs, with particular emphasis on the role of genetic testing in risk stratification and management.

## 1. Introduction

Heritable thoracic aortic disease (HTAD) comprises a large and heterogenous group of disorders characterized by aortic events, mainly represented by aneurysm or dissection [1]. The early identification of inherited diseases associated with increased risk for acute aortic events is crucial to perform tailored management strategies.

The clinical course of patients with HTAD is extremely variable, ranging from those with early-onset and aggressive to those with late-onset, indolent aortic disease. In addition, the age at onset of acute aortic syndromes can be variable among patients who carry the same pathogenic mutation or different pathogenic mutation in the same gene [1,2].

According to the presence or absence of extra-aortic features, HTADs are classified as syndromic and non-syndromic, respectively [2]. Since 20–25% of patients with non-syndromic HTAD present a family history of aortic disease, the clinical evaluation of the family members of the proband is required to differentiate familial from sporadic cases [3].

**Figure 1:**
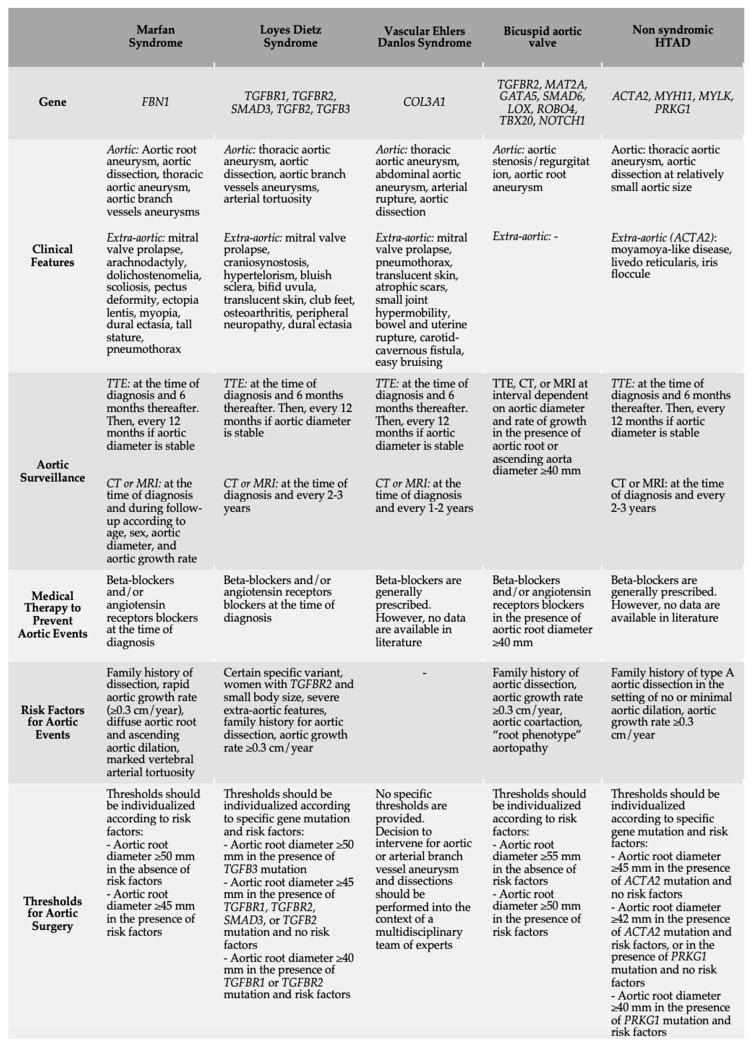
Clinical and genetic characteristics, risk factors for acute aortic events, surveillance, medical therapy, and recommendations for aortic surgery according to each HTAD. Aortic surveillance and thresholds for aortic surgery are those proposed by the 2022 American College of Cardiology/American Heart Association guidelines for the diagnosis and management of aortic disease [5]. Abbreviations: CT, computed tomography; MRI, magnetic resonance imaging; HTAD, heritable thoracic aortic disease; TTE, transthoracic echocardiography.

Identifying a genetic cause of HTAD has a significant impact on patients’ management, since the different conditions significantly differ with respect to natural history and treatment strategies [4,5] (Figure 1). In addition, genetic testing may guide family screening, leading to the identification of other family members at risk [1]. Furthermore, the early detection of carriers (i.e., individuals who have inherited the gene mutation but who do not express the clinical phenotype) can allow individualized aortic surveillance [6,7]. For example, patients with pathogenic mutation in *TFGBR1*, *TGFBR2*, and those with specific mutation in *ACTA2* (e.g., Arg179) exhibited a significant burden of aortic disease in childhood, with the recommendation to start clinical and echocardiographic follow-up in the first decade of life [7]. Indeed, the TGF-β pathway has a crucial role in regulating vascular remodeling and effects on extracellular matrix synthesis and degradation [8]. Thus, pathogenic mutations in *TGFBR1* and *TGFBR2* lead to a pathological shift towards increased extracellular matrix degradations, finally responsible for aneurysm formation and increased susceptibility to rupture [9].

The prognosis in all HTAD is determined by the progressive dilation of the aorta, potentially leading to acute aortic events, such as dissection or rupture [4]. These events generally involve the ascending aorta, although the involvement of other districts of the aorta or peripheral vessels may occur [6].

Of importance, the prognosis varies according to the underlying genetic mutations [7]. Unfortunately, we still do not have accurate models for predicting adverse outcomes in patients with specific gene mutations. However, with the accumulation of data regarding the long-term outcome of each specific gene or genetic variant, it will be possible to develop individualized surveillance programs and risk stratification scores. Hopefully, this will be responsible for an improvement of outcomes of both probands and family members of patients with HTADs.

This review aims to describe the clinical characteristics and natural history of the most common HTADs, with particular emphasis on the role of genetic testing in risk stratification and management.

## 2. Methods

A search of the English language literature was performed using PubMed up to December 2022 on the clinical features, genetics, pathophysiology, diagnosis, and treatment of HTAD.

The terms heritable thoracic aortic disease, inherited aortic disease, genetic aortic disease, Marfan syndrome, Ehlers Danlos syndrome, Loyes Dietz syndrome, bicuspid aortic valve, *FBN1*, *COL3A1*, *TGFBR1*, *TGFBR2*, *ACTA2*, *MYH11*, *PRKG1*, and *MYLK* individually combined with either aortic dissection, aortic dilation, aortic event, clinical characteristics, genotype, gene testing, genetic testing, or phenotype were used.

Observational studies, case reports, reviews, position papers, and guidelines were included in our search. References were carefully evaluated for missing publications. Information on genetic mutations, pathophysiology, clinical characteristics, diagnostic and therapeutic strategies were extracted from the literature.

## 3. Syndromic HTAD

Syndromic HTADs are characterized by the presence of aortic and extra-aortic involvement. These conditions can be classified according to the type of gene mutation: HTAD related to genes encoding for components of the extracellular matrix (e.g., Marfan syndrome, Vascular Ehlers Danlos syndrome); and HTAD related to genes encoding for components of the transforming growth factor beta (TGF-β) (e.g., Loyes Dietz Syndrome) [1].

### 3.1. HTAD Related to Genes Encoding for Components of the Extracellular Matrix

#### 3.1.1. Marfan Syndrome

Marfan syndrome (MFS) is a rare connective disorder, with a prevalence of 1–5 cases among 10,000 people [10]. It is inherited as an autosomal dominant trait caused by a disease-causing mutation in *FBN1* (encoding for fibrillin-1) in more than 95% of cases [10].

Clinical manifestations include cardiac, skeletal, and ocular involvement. Mitral valve prolapse (MVP) is the most prevalent valvular abnormality, identified in more than half of patients [10]. In addition, aortic regurgitation, secondary to aortic root dilation or caused by structurally abnormal valves, is also common, ranging from 14 to 44% of cases [11]. Bicuspid aortic valve (BAV) is rarely observed in patients with MFS, but when present, it is associated with a worse clinical phenotype [12].

The prognosis mainly depends on the aortic involvement. Aortic dissection represents the leading cause of mortality in patients with MFS [1]. In particular, the aortic component more prone to aortic dilation is the aortic root, with the possible development of aortic aneurysm and associated risk of type A dissection or rupture. Moreover, about 10–20% of patients may present the involvement of the descending thoracic or abdominal aorta, eventually leading to type B dissection [13]. Aortic branch aneurysms are present in one-quarter of patients with MFS, are related to age and aortic dilation, and independently predict the need for aortic surgery [6].

Several disease-causing mutations in *FBN1* have been associated with isolated thoracic aortic disease, without the presence of systemic features.

The diagnosis of MFS is based on clinical and genetic features, according to the revised Ghent criteria [14]. Diagnosis is established in patients with *FBN1* pathogenic variant known to be associated with MFS and one of the following additional criteria: aortic root enlargement (defined as a Z-score ≥2); or ectopia lentis. Moreover, the diagnosis should be suspected in individuals with suggestive clinical findings (e.g., pectus carinatum or excavatum, pes planus, pneumothorax, dural ectasia, scoliosis, myopia, MVP).

The molecular genetic testing approach varies according to the clinical presentation. In patients with clinical features strongly suggestive of MFS, single-gene testing is recommended. On the other hand, when the phenotype is clinically indistinguishable from other inherited disorders, a multigene panel (including *FBN1* and other genes associated with HTAD) should be considered.

The main aim of the treatment in patients with MFS is to prevent the development/progression of aortic aneurysms and their dissection/rupture. For this purpose, beta-blockers and angiotensin receptor blockers (ARBs) are commonly prescribed. According to the 2020 European Society of Cardiology (ESC) guidelines for the management of adult congenital heart disease [6], beta-blockers represent the mainstay for medical treatment for MFS/HTAD patients, given their effectiveness in reducing wall shear stress and aortic growth rate [15]. However, ARBs have been shown to have a similar or even higher effect than beta-blockers in several trials and should be considered as an alternative to beta-blockers [16,17]. In addition, a recent individual patient meta-analysis showed that ARBs reduced the rate of increase of the aortic root Z-score by about one-half, including among patients taking beta-blockers [18]. Thus, the authors concluded that, assuming additivity, the combination therapy with both ARBs and beta-blockers would provide a more significant reduction in the rate of aortic enlargement than treatment alone [18]. Thus, medical treatment, if started at the diagnosis, would be expected to delay the need for aortic surgery.

This recent evidence was incorporated into the recent 2022 American College of Cardiology/American Heart Association (ACC/AHA) guidelines for the diagnosis and management of aortic disease, which recommends the use of both a beta-blocker and an ARB in maximally tolerated doses to reduce the rate of aortic dilation [5].

The identification of *FBN1* mutation associated with MFS has significant management implications. For example, aortic root surgery is recommended at lower aortic diameter thresholds in patients with MFS compared with those recommended for the general population. In particular, both the 2020 ESC guidelines for the management of adult congenital heart disease [6] and the 2022 ACC/AHA guidelines for the diagnosis and management of aortic disease [5] recommend to perform surgery in patients with MFS with aortic root disease with a maximal aortic sinus diameter ≥50 mm, or ≥45 mm in the presence of additional risk factors (Figure 1). 

In patients with MFS, intense physical activity is discouraged due to the risk of progression of aortic dilation and aortic rupture [19,20]. The 2020 ESC guidelines of sport cardiology provide specific recommendations in terms of physical activity prescription in patients with MFS or other HTADs [19]. In detail, patients with MFS without aortic dilatation (i.e., with a maximal aortic diameter <40 mm) are considered at low to intermediate risk and are recommended to avoid high and very high intensity exercise, contact and power disciplines, and to clinical re-evaluate the patient every one to two years. Patients with moderate aortic dilation (i.e., with a maximal aortic diameter of 40–45 mm) are considered at intermediate risk, and in these individuals only skilled, mixed or endurance disciplines at low intensity are permitted with a periodic follow-up every six to twelve months. Finally, patients with severe aortic dilatation (i.e., with a maximal aortic diameter >45 mm) are considered at high risk and all sports are contraindicated.

#### 3.1.2. Vascular Ehlers Danlos Syndrome

Vascular Ehlers Danlos syndrome (vEDS) is a multisystemic disorder inherited as an autosomal dominant trait caused by a pathogenic variant in the *COL3A1* gene [21]. COL3A1 gene encodes for a component of type III collagen, called pro-α1 chain. Three α1 chain are folded into a triple helix, which represents the structural domain of the type III collagen. Mutations in COL3A1 are responsible for the production of mutant pro-α1 chain that are either degraded or accumulated into intracellular cytoplasm, leading to a severe reduction in type III collagen [22].

The diagnosis should be suspected in patients with major or minor diagnostic criteria (e.g., arterial aneurysms, intestinal rupture, uterine rupture during pregnancy, family history of vEDS) according to the diagnostic criteria proposed by Malfait et al. [21]. Subsequently, genetic testing with the demonstration of disease-causing mutation in *COL3A1* is required to confirm the diagnosis.

Severe disease complications generally appear during childhood and could repeat in an unpredictable fashion [23]. Vascular abnormalities, including aneurysms, dissection, and rupture of major and minor arteries, are the most common disease presentations, followed by gastrointestinal rupture. It has been estimated that the life expectancy of patients with vEDS is significantly reduced, with a median of 51 years of age [23,24].

Data on medical therapy with effectiveness in reducing the risk for vascular complications are scant and limited to small studies investigating the role of beta-blockers. A multicenter, randomized, open trial including 53 patients with clinical vEDS investigated the role of celiprolol in preventing arterial events, including fatal and non-fatal rupture or dissection [25]. After a mean follow-up of 47 months, the trial was stopped early for treatment benefit. In particular, arterial events occurred in 20% of patients on celiprolol and in 50% of patients on placebo, thus suggesting that celiprolol might be the treatment of choice for vEDS patients. The potential role of celiprolol in reducing the risk for arterial events was confirmed by a large cohort study, which showed that patients treated with celiprolol had better survival than those without, and that the overall reduction in mortality was dose-dependent [26].

Unfortunately, no guidelines or consensus expert documents are available for patients with vEDS. Thus, while there is agreement that vascular dissection and rapid arterial aneurysm growth represent clear indications for urgent surgery, the decision about the timing and approach of elective vascular procedures is not well established. 

In patients with vEDS, surgical repair is associated with increased risk for severe complications related to vascular fragility. Thus, each surgical decision should consider the patient’s individual characteristics, discussed by a multidisciplinary team of experts, and defined after a comprehensive risk/benefit evaluation. 

### 3.2. HTAD Related to Genes Encoding Component of the TGF-β

#### Loeys–Dietz Syndrome

Loeys–Dietz syndrome (LDS) is an autosomal dominant disease caused by a heterozygous mutation in transforming growth factor receptors 1 and 2 (*TGFBR1* and *TGFBR2*) genes. Bart Loeys and Harry Dietz described it for the first time as they observed a complex phenotype in 52 families, mainly characterized by a triad of aortic aneurysms and generalized arterial tortuosity, hypertelorism, and bifid uvula and/or cleft palate [27]. Since then, mutations of *SMAD3*, *TGFB2* and *TGFB3*, associated with their progression to aggressive vascular diseases, have been included under the definition of LDS [28,29]. To date, six subtypes of LDS are currently described.

In addition to the triad of symptoms mentioned above, clinical manifestations of LDS may affect the skeletal system, similarly to Marfan syndrome (pectus deformities, camptodactyly, joint hyperextension, low bone mineral density) [30,31,32]. In contrast, the different cutaneous phenotype (i.e., LDS is characterized by thin and translucent skin with visible veins) is crucial for the differential diagnosis between these connective tissue diseases [33]. Cardiac features include MVP with mild-to-severe mitral regurgitation and congenital heart diseases (such as BAV, atrial septal defect, or a patent ductus arteriosus), which are more frequently reported in LDS individuals than in the general population. Inflammatory and immunological dysregulations and association to inflammatory bowel disease, allergy or osteoarthritis have frequently been observed in such patients [34]. Vascular anomalies are the most common and concerning manifestations of LDS. Since the whole vascular system may be affected by connective tissue disease, arterial tortuosity and aneurysms must be sought in the entire body. Intracranial vessels often show increased tortuosity and aneurysmal dilations, and tortuosity index (TI) could be helpful in the differential diagnosis between Marfan syndrome and LDS [35].

LDS is considered a rapidly progressive aortic aneurysmal disease, requiring close monitoring. Aneurysms are most commonly found at the aortic root, while ascending and descending aorta are less commonly affected [36]. The growth rate is much faster than sporadic aortic aneurysms (increase in diameter >1 cm/y), resulting in the mean age of death of 26 years. Aortic dissection has even been reported in infants at smaller aortic diameters than sporadic aortic aneurysms (average of 4,6 cm). Thus, the 2022 ACC/AHA guidelines for aortic diseases recommend whole body imaging (from cerebral circulation to pelvis) in all patients with LDS at diagnosis and six monthly afterwards to establish if enlargement is occurring [5].

Since the increase in TGF-β and SMAD2 signaling induced by angiotensin receptors has been evidenced in mouse models [37], prophylactic medical treatment with ARB has been proposed. Moreover, pilot studies reported improved Pulse Wave Velocity and arterial stiffness indexes in patients treated with losartan [38]. Beta-blockers have also been tested, although no effect has been observed on aortic wall architecture in mouse models [37]. A recent study on smooth muscular cells also showed that combination treatment with activin A and rapamycin may increase contractile protein levels in mutated TGFBR1 cells, and this may represent a therapeutic possibility in the future [39].

Currently, 2022 ACC/AHA guidelines for aortic disease recommend starting beta-blockers and/or ARBs at the time of diagnosis in order to reduce aortic growth rate and reduce the occurrence of aortic events [5].

Prophylactic surgery should be performed to prevent the rupture of aneurysms. Patients with pathogenic variants in *TGFBR1* or *TGFBR2* exhibit a more aggressive clinical course than patients with MFS, leading to more aggressive aortic disease management recommendations [5]. Thus, according to the 2022 ACC/AHA guidelines, the thresholds for aortic surgery should be individualized according to the type of mutations and presence of additional risk factors [5]. For example, patients with mutations in *TGFBR1* and *TGFBR2* with increased risk for aortic events (e.g., family history of aortic dissection, rapid aortic growth) should undergo aortic surgery when their aortic root diameter is ≥40 mm [5]. A higher risk of complications has been associated with *TGFBR2* mutation and extra aortic features of LDS, thus it has been proposed to offer the possibility of prophylactic surgery even to patients with diameters <40 mm [40]. In addition, a recent study demonstrates better-than-expected survival in patients with *TGFBR1* or *TGFBR2* gene mutation [41]. Therefore, further lowering the threshold may not be necessary in such cases.

In children, according to AHA Guidelines, surgical intervention should be considered once the aortic diameter surpasses the 99th percentile for age and body surface area and as soon as the aortic valve annulus reaches 18–20 mm. In contrast, MacCarrick et al. suggest delaying surgery until the aortic annulus is 2.0–2.2 cm in order to accommodate adult-sized graft, or waiting until it reaches 4 cm of diameter in children with slow progressive enlargement. In addition, a rapidly expanding aorta (0.5 cm over 1 year), severe craniofacial features and a family history of aggressive aortic disease should be taken under consideration for earlier surgical intervention [33].

Aortic valve-sparing root replacement (VSRR) is the most recommended surgery procedure in patients with LDS because the mortality rate is low, and post-operative anticoagulation therapy is not required. Bentall procedure (i.e., the replacement of aortic root and the aortic valve with a composite aortic valve graft) has also been successfully performed, and is taken under consideration by AHA Guidelines. Endovascular aneurysm repair (TEVAR) is not recommended in these patients because progressive aneurysm development may generate a false lumen and result in graft failure. It has been suggested to perform endovascular repair in localized segments of the aorta when proximal and distal landing zones lie within a surgical graft or in case of peripheral aneurysm [42]. The introduction of hybrid procedures may lead to new therapeutic chances in the future.

## 4. Non-Syndromic HTAD

### 4.1. Bicuspid Aortic Valve

Bicuspid aortic valve (BAV) is a frequent congenital heart defect in the general population with an overall incidence of 2% in the Western world. This valvulo-aortopathy presents the fusion of the cusps of the aortic valve, which is normally a trileaflet valve, resulting in only two commissures delimiting two valve cusps [4]. Different fusion patterns and several classifications have been proposed to describe BAV. However, a consistent description of valve morphology and specific associated phenotypes divides patients with bicuspid aortic valve into three main groups [43]: Fused BAV (90–95% of cases) with two cusps of different size, a symmetrical or asymmetrical non-fused commissural angle and a congenital fibrous ridge called raphe, usually well visible; two sinus BAV (5–7% of cases) with two cusps of the same size and a symmetrical non-fused commissural angle without raphe; partial-fusion BAV, a mild form with three cusps usually symmetrical and three commissures where two are normal and the third is partially fused. A mini raphe can be present [44].

The specific phenotypes associated with these patterns of fusion are right–left cusp fusion (70–80% of cases), right non-coronaric cusp fusion, and left non-coronaric fusion for “fused BAV pattern”; laterolateral and anteroposterior phenotypes for “2 sinus BAV pattern” [43].

The presence of BAV in syndromic patients, the association with other congenital defects and the familial occurrence in 5–10% of first-degree relatives suggest a genetic etiology [45]. Some of the main causative mutations involve *NOTCH1*, GATA, *SMAD*, and *ACTA2* [46]. However, these genetic variants can be found in <5% of all BAV patients. For this reason, genetic testing may be considered only in specific familial cases [4].

Principal clinical manifestations of BAV are related with valvular disfunction, aortopathy and endocarditis [47]. While the risk of aortic valvular regurgitation is approximately 30%, rising to 50% 25 years after BAV diagnosis, aortic stenosis is much more common. Surgical correction is the gold standard and TAVR can be an alternative for patients at high surgical risk [48]. The presence of a raphe is associated with progression of valvular dysfunction (particularly AS) and the need for future valvular correction [47,48]. Moreover, aortic regurgitation in BAV patients is associated with more extended and severe myocardial fibrosis than in trileaflet valve patients. Shear stress, abnormally distributed among the asymmetric cusps, promotes a turbulent and eccentric flow responsible for interstitial fibrosis and left ventricular remodeling, especially in basal segments. For this reason, patients with BAV have a higher degree of left ventricular remodelling, higher prevalence of myocardial fibrosis, and an overall worse prognosis than the general population [49].

Over half of BAV patients develop aortic dilation which can progress to dissection and rupture, increasing the risk of sudden death [43,44]. From a pathophysiological standpoint, the aortic root architecture is crucial to maintaining a sufficient diastolic cusp coaptation, preventing progression to valvular insufficiency. Since survival in patients with BAV is similar to the general population, the elective surgical cut-off for aortic dilatation is the same (≥55 mm). However, the presence of associated risk factors (e.g., severe valvular regurgitation, aortic coarctation, family history of aortic dissection, aortic growth rate ≥3 mm/year) increases the risk of vascular complications and reduces the threshold to ≥50 mm [6,43]. Data regarding pregnant women with BAV and aortopathy is limited and lacks consistency. Current ESC guidelines for the management of cardiovascular diseases during pregnancy recommend against pregnancy when the aortic diameter is ≥50 mm [50].

Recognizing the BAV pattern of fusion has clinical relevance because its association with specific valvular and aortic phenotypes can predict different clinical courses. Patients with right non-coronary cusp fusion present more often with valvular stenosis and ascending aortic dilation, while patients with aortic root phenotype often present a right–left cusp fusion associated with mild to severe valvular regurgitation [43]. 

In terms of prognosis, BAV patients can be categorized into three groups: complex valvulo-aortopathy, where BAV is usually associated with genetic syndromes (e.g., Marfan Syndrome, Loeys–Dietz Syndrome, Turner Syndrome) or congenital heart disease (e.g., aortic coarctation, ventricular septal defect, atrial septal defect, coronary anomalies, patent ductus arteriosus and mitral valve prolapse) [7]; typical valvulo-aortopathy, most common with BAV presentation; uncomplicated BAV, a silent condition with mild or no progressive aortopathy, usually diagnosed incidentally [43].

Patients with complex and typical phenotypes are at higher risk of developing endocarditis and disease progression. The prognostic presentation of these groups is summarized in Table 1.

### 4.2. Other Non-Syndromic HTADs

Mutations in genes encoding for protein involved in smooth muscle contraction (SMC) are responsible for most non-syndromic HTADs. These genes include *ACTA2*, *MYH11*, *PRKG1*, and *MYLK* [51]. 

Mutations in *ACTA2* are the most common cause of non-syndromic HTAD [52], responsible for 12% to 21% of familiar HTADs without features of connective tissue disorders. The *ACTA2* gene encodes for the smooth muscle cell specific isoform of alpha-actin. This condition is inherited as an autosomal dominant trait and is characterized by aortic aneurysm, and occasional extravascular traits (e.g., skin and ocular abnormalities). In particular, the mutation involving the Arg179 residue of *ACTA2* is responsible for the multisystem smooth muscle dysfunction syndrome, a systemic disease characterized by aortic and cerebrovascular disease, fixed mydriatic pupils, hypotonic bladder, intestinal hypoperistalsis, pulmonary hypertension, and brain abnormalities [52].

The risk of acute aortic events differs according to the gene involved and to the specific type of mutation. Recently, the Montacino Aortic Consortium (MAC) was assembled with the aim of defining the natural history of and the risk for adverse outcomes in patients carrying disease-causing mutations associated with HTADs, representing a multicenter effort to define the full spectrum of these complex conditions [7]. They found that the risk of aortic events was different among patients carrying mutations in different SMC genes. In particular, patients carrying *PRKG1* pathogenic variants experienced a higher rate of aortic events compared with those with *ACTA2* and *MYLK* mutations. In addition, it was found that the cumulative risk of composite aortic events at 25 years was 15–27% for *ACTA2* and *PRKG1*, while the incidence was significantly lower in patients with different gene mutations (i.e., *MYLK*, *SMAD3*, *TGFB2*) [7]. Of relevance, the cumulative risk for composite events at 65 years was 70%, showing that most patients with isolated HTADs associated with a mutation in the SMC gene will experience an aortic event during their lifetime.

Furthermore, a large cohort study evaluating 277 individuals carrying an *ACTA2* mutation showed that half of these patients experienced an aortic event, mainly represented by thoracic aortic dissection (mainly type A dissection). In addition, the type of gene mutation significantly influenced the outcome. After adjustment for sex, race, and intrafamiliar correlation, the mutation involving the Arg179 or Arg258 residues of *ACTA2* was associated with significantly increased risk for aortic events [52]. 

Since patients with mutations in the ACTA2 gene do not present extra-aortic features, they are often diagnosed on presentation with aortic events. Therefore, any patients with acute aortic dissection without systemic features suggestive of specific syndromes and with family history of aortic events should raise the suspicion for an underlying *ACTA2* gene or other SMC genes.

Genetic testing for *ACTA2* mutations should be considered in patients with one or more of the following conditions: early onset thoracic aortic dissection (type A or B dissection); family history of acute aortic events; peripartum aortic dissection; clinical features associated with *ACTA2* mutation (e.g., early-onset stroke, livedo reticularis, iris floccule, or other extra-aortic features associated with multisystem smooth muscle dysfunction syndrome).

Due to the increased risk of aortic events in this special population, recommendations for elective aortic root replacement suggest considering surgery at lower thresholds compared with those recommended for the general population or with other inherited conditions (e.g., MFS) [53]. The 2022 ACC/AHA guidelines recommend surgery in patients with a maximal aortic diameter ≥45 mm and *ACTA2* mutation, or lower in patients with risk factors or specific gene mutations [5]. On the other hand, no specific guidelines are provided by the ESC guidelines. Thus, in the absence of specific recommendations and large studies investigating the natural history of these rare conditions, the management strategy should be individualized for each single patient. Several factors should be considered other than the maximal aortic diameter, such as the family history for aortic events, the annual growth rate, the desire for pregnancy, the body surface area, the presence of uncontrolled hypertension.

In these patients, a tailored aortic surveillance, intensive blood pressure monitoring and treatment, the avoidance of specific physical activities, and a timely surgical aortic repair are required to reduce the risk of aortic events and death associated with *ACTA2* mutations.

Similarly to patients with MFS, patients with isolated HTADs associated with mutation in the SMC genes should avoid contact or power sports, and high intensity activities. In addition, specific recommendations should be applied according to the presence of significant aortic dilation or valvular disease [19].

## 5. Conclusions

HTADs are a large and heterogenous group of disorders characterized by the occurrence of aortic events, mainly aneurysms and dissection. Genetic diagnosis has a significant impact on patients’ management since the different conditions significantly differ with respect to natural history and treatment strategies. Several factors enhance the complexity of the decision-making in patients with syndromic or isolated HTADs (e.g., the presence of interactions between genetic and environmental factors, the possible cumulative impact of coexisting variance of uncertain significance or gene modifiers, and the effect of environmental factors, such as hypertension).

## Figures and Tables

**Table 1:** Clinical course of patients with BAV according to the pattern type.

BAV Type	Presentation	Valve Disfunction	Aortopathy	Main Complications	Prognosis
Complex	Genetic syndromes and congenital heart lesions	Early and rapid	Early and rapid	Aortic dissection Endocarditis	Life expectancy may be reduced
Typical	Isolated BAV	Progressive	Progressive	Aortic dissection Endocarditis	Life expectancy usually preserved
Uncomplicated/ Undiagnosed	Isolated BAV	Mild and/or not progressive	Mild and/or not progressive	Typically silent condition	Excellent

## Data Availability

The data presented in this study are contained within the article.
